# Pregnancy Outcomes in Women With SARS-CoV-2 Infection During the First and Second Waves of the COVID-19 Pandemic in a Tertiary Care Hospital in Ayodhya, Uttar Pradesh, India: A Comparative Study

**DOI:** 10.7759/cureus.34969

**Published:** 2023-02-14

**Authors:** Diksha Ambedkar, Yogesh Yadav, Pawan Dubey, Vijay Kumar, Rina Sharma, Charu Mishra

**Affiliations:** 1 Obstetrics and Gynaecology, Rajarshi Dashrath Autonomous State Medical College, Ayodhya, IND; 2 Pathology, Rajarshi Dashrath Autonomous State Medical College, Ayodhya, IND; 3 Biostatistics and Epidemiology, Autonomous State Medical College Basti, Rampur, IND; 4 Plastic Surgery, King George's Medical University, Lucknow, IND; 5 Physiology, Madhav Prasad Tripathi Medical College Siddharthnagar, Basadiliya, IND

**Keywords:** pandemic, pregnancy, long term sequelae, feto-maternal outcome, severity

## Abstract

Introduction

Pregnancy is an altered immunological state and not necessarily an immune-compromised state. These immune changes subject pregnant women to increased susceptibility to infection. During the coronavirus disease 2019 (COVID-19) pandemic, pregnant women were more susceptible to serious illness for reasons other than their immune response. The purpose of this study was to compare the feto-maternal outcome (morbidity and mortality) in relation to pre-existing maternal co-morbidities, severe acute respiratory syndrome coronavirus 2 (SARS-CoV-2) infection-related disease severity, and its impact on the mode of delivery and long-term sequelae in pregnant women in the first and second waves of the COVID-19 pandemic.

Materials and methods

This was a hospital-based comparative study carried out on 101 pregnant patients during the first wave (April 2020 to December 2020) and 22 patients in the second wave (March 2021 to July 2021) of the COVID-19 pandemic, in Rajashri Dashrath Autonomous State Medical College, Ayodhya, India. All pregnant women with COVID-19 in the first and second waves were included. Non-pregnant patients with COVID-19 infection, pregnant patients lost to follow-up, pregnant patients without COVID-19 infection, and patients in the puerperal period were excluded.

Results

Seventy-three (72.27%) patients in the first wave and 12 (54.54%) in the second wave were asymptomatic. Those with mild disease numbered 20 (25.74%) in the first wave and six (27.27%) in the second wave. Disease severity was more in the second wave, that is four (18.18%) as compared to one (0.99%) in the first wave. Severe anemia was the most common co-morbidity associated with both first (n=4, 3.96%) and second (n=5, 22.72%) waves. Four (6.45%) spontaneous abortions occurred in the first wave as compared to three (20%) in the second wave. Intensive care unit (ICU) admission was more in the second wave (n=4, 26.66%) as compared to the first wave (n=1, 1.61%). Two (13.33%) maternal deaths occurred in the second wave and none in the first wave. Cesarean sections in both the first and second waves were performed for obstetric indications only. No newborns tested positive in the COVID-19 reverse transcription-polymerase chain reaction (RT-PCR) in the first and second waves at the time of birth; however, three (4.83%) tested positive on day five of birth in the first wave. Fever was the most common presentation in newborns; seven (11.26%) in the first wave and three (20%) in the second wave. No neonatal death occurred in the first or second waves. No congenital anomalies were noted in the first or second waves of COVID-19.

Conclusion

In this study, we found that the maximum number of COVID-19-positive pregnant patients in both the first and second waves of COVID-19 were either asymptomatic or had mild infections. Second-wave infection was more lethal as compared to the first wave in terms of adverse maternal as well as fetal outcomes. No gestational age was an exception to the severity of disease and its adverse feto-maternal outcome. In our study, maternal co-morbidities did not impact the overall outcome. All cesarean sections were performed for indications other than COVID-19 infection. Long-term sequelae associated with COVID-19 were seen in both groups but more so in the second wave. No long-term sequelae like congenital anomalies in the babies were associated with COVID-19 either in the first or second wave.

## Introduction

Worldwide, severe acute respiratory syndrome coronavirus (SARS-CoV) has been affecting humankind time and again with the various strains causing severe acute respiratory syndrome (SARS), Middle East respiratory syndrome (MERS) [1,2], and recently coronavirus disease 2019 (COVID-19), which was a pivot worldwide when it first emerged as a disease of concern in December 2019 and evolved as a pandemic by March 2020 [3]. Ever since, the virus has been mutating so as to affect populations with varying severities and following almost similar trends worldwide. Regarding COVID-19 in pregnancy, several studies have been conducted in relation to feto-maternal outcomes, and mixed opinions have been obtained. While some studies suggest a significant adverse impact of the disease in a pregnant patient [4-6], others deduced that there was no such difference when compared to the normal population [7].

Pregnancy is an immune condition unlike others. It is suggested that the maternal immune system is a variable condition wherein during the first trimester, the patient is in a pro-inflammatory state, in the second trimester an anti-inflammatory environment exists, while in the third trimester again there is a pro-inflammatory state [8].

Some studies are suggestive of a possible association between disease severity and medical co-morbidities in pregnancy. Evidence has shown that pregnant patients are vulnerable to adverse outcomes with COVID-19 [7,9]. Pregnant patients are at an increased risk of complications that affect both mother and baby, and there is an increased chance of preterm or stillbirth [7]. SARS infection during pregnancy has been associated with a higher rate of spontaneous abortion, preterm birth, and intrauterine growth restriction (IUGR), in various studies [8].

The emergence of the Delta variant in the second wave of COVID-19 was associated with a greater risk of ICU admissions, increased medical interventions like invasive ventilation and extracorporeal membrane oxygenation (ECMO), and increased risk of maternal and fetal mortality [7].

This study was an attempt to put forth our experience regarding disease severity and possible feto-maternal outcomes in the first and second waves of COVID-19 in a tertiary hospital catering to rural and urban populations and at the same time to identify an impact in relation to the associated co-morbidities and any impact on the mode of delivery in these patients. We also intended to determine any long-term sequelae in the first and second wave groups.

## Materials and methods

This was a prospective study conducted from April 2020 to 2022 at the associated hospital of Rajashri Dashrath Autonomous State Medical College, Ayodhya, India. A total of 123 pregnant women were enrolled in the study. Of these, 101 patients were from the first wave and 22 from the second wave of the COVID-19 pandemic. This was a comparative study in relation to the feto-maternal outcome in the two groups. Outcomes were also studied in relation to the disease severity, association with pre-existing comorbidities, impact on mode of delivery, and long-term sequelae in the two groups. All COVID-19-positive pregnant patients irrespective of gestational age, parity, and any associated co-morbidities were included in the study. Pregnant patients lost to follow-up and with COVID-19-negative status, patients in puerperal period with COVID-19, and non-pregnant female patients were excluded from the study. The study was approved by the Institutional Ethics Committee of Rajarshi Dashrath Autonomous State Medical College, Ayodhya, India (Approval number: RDASMC/IEC/2020/04) and informed consent was taken from participants or their attendants.

Patients were categorized on the basis of clinical presentation and grouped accordingly into asymptomatic, mild (uncomplicated upper respiratory tract infection with oxygen saturation (SpO2) of approximately 94% in room air, respiratory rate (RR) of 24/minute, and no evidence of hypoxemia or breathlessness), moderate (pneumonia with no signs of severe disease, SpO2 94-90% in room air, RR 24-30/min), and severe (severe pneumonia, SpO2 < 90%, RR >30/min) disease, critically ill (acute respiratory distress syndrome (ARDS) and septic shock) [10].

Data were obtained from the patients admitted to the labor room who were referred from level one hospitals to our center, which was designated as a level two COVID-19-dedicated hospital. All patients were followed up for a period of one year from the time of infection to know the possible feto-maternal outcome and to find out any long-term sequelae in the mother and the baby. Rooming in of the babies was done for those subjects who delivered at our center while taking all precautions like hand washing, wearing masks before breastfeeding, and keeping the baby at a distance from the mother when not breastfeeding. One apparently healthy caregiver accompanying the patient was permitted to stay with the patient taking all due precautions and COVID-19 testing with RT- PCR was performed on the babies on the first and fifth day of delivery and at the time of discharge and the caregivers were tested on the fifth day of contact with the patient and at the time of discharge. Those babies and caregivers who turned SARS-CoV-2 positive during the hospital stay were notified and managed accordingly.

Statistical analysis was done with IBM SPSS Statistics for Windows, Version 27.0 (Released 2020; IBM Corp., Armonk, New York, United States). Categorical variables were expressed as the number and percentage of patients and were compared across the groups using Pearson’s Chi-Square test for independence of attributes or Fisher’s Exact test as appropriate. An alpha level of 5% was taken, and a p-value less than 0.05 was considered statistically significant throughout the study.

## Results

Out of the total 1443 COVID-19-positive admissions in the first wave, 101 (6.99%) were pregnant females. In the second wave, out of the total 1036 admissions, 22 (2.12%) were pregnant females. The total number of admissions of COVID-19 patients in the general population was almost the same in both the first 1342 (93%) and the second 1014 (97.87%) waves; however, in pregnant females. the number of admissions was much higher in the first wave as compared to the second wave of the COVID-19 pandemic (Table 1).

**Table 1:** Number of patients admitted COVID-19: coronavirus disease 2019

	First Wave of COVID-19	Second wave of COVID-19
Total admission in hospital of COVID-19-positive patients	1443	1036
General patients	1342 (93%)	1014 (97.87%)
Pregnant patients	101 (6.99%)	22 (2.12%)

The number of admissions with pregnancy-induced hypertension was one (0.99%) in the first wave and one (4.5%) in the second wave of COVID-19. One (4.5%) case of antepartum eclampsia was notified and brought to our center in the second wave but none in the first wave. One (0.99%) patient with gestational diabetes was admitted in the first wave but none in the second wave. Severe anemia was significantly associated in both waves of COVID-19 (p=0.002) with four (3.96%) and five (22.72%) in the first and second waves, respectively. Two (1.98%) cases of hypothyroidism associated with pregnancy were admitted in the first wave but none in the second wave. Cholestasis of pregnancy was seen in one (0.99%) in the first wave and one (4.5%) in the second wave. One (0.99%) case of pregnancy with epilepsy with controlled seizures was reported in the first wave of COVID-19 and none in the second wave. One (0.99%) HIV patient on antiretroviral therapy (ART) with normal clusters of differentiation 4 (CD4) count without evidence of AIDS was admitted to the labor room in the first wave of COVID-19 and none in the second wave (Table 2).

**Table 2:** Associated co-morbidities in pregnant females with COVID-19 Note: * significant at α=5% COVID-19: coronavirus disease 2019

Comorbidity	First wave of COVID-19 (n=101)	Second wave of COVID-19 (n=22)	P-value
Hypertension	1 (0.99%)	1 (4.5%)	0.234
Eclampsia	0 (0%)	1 (4.5%)	0.031*
Diabetes mellitus	1 (0.99%)	0 (0%)	0.638
Severe anemia	4 (3.96%)	5 (22.72%)	0.002*
Hypothyroidism	2 (1.98%)	0 (0%)	0.502
Jaundice/ Cholestasis	1 (0.99%)	1 (4.5%)	0.234
Epilepsy	1 (0.99%)	0 (0%)	0.638
HIV	1 (0.99%)	0 (0%)	0.638

The total number of infected individuals with COVID-19 disease was higher in the first wave as compared to the second wave, and of these, the number of asymptomatic cases was higher in the first wave (n=73, 72.27%) as compared to the second wave (n=12, 54.54%). The percentage of patients with mild disease was almost similar in the first (n=20, 25.74%) and second (n=6, 27.27%) waves of COVID-19. One (0.99%) case with moderate disease was reported in the first wave but none in the second wave. The number of patients with severe disease and critical illness was four (18.18%) in the second wave as compared to one (0.99%) in the first wave. Disease severity was significantly associated with the second wave of COVID-19 at 5% level of significance (Table 3).

**Table 3:** Disease severity at presentation Note: * significant at α=5% COVID-19: coronavirus disease 2019

Symptoms severity	First wave of COVID-19 (n=101)	Second wave of COVID-19 (n=22)	Test statistics
Asymptomatic	73 (72.27%)	12 (54.54%)	14.19*
Mild	26 (25.74%)	6 (27.27%)	
Moderate	1 (0.99%)	0 (0%)	
Severe/Critically Ill	1 (0.99%)	4 (18.18%)	

Four (6.45%) cases of spontaneous abortion were recorded in the first wave. The incidence of spontaneous abortions was higher in the second wave (n=3, 20%). The incidence of preterm labor was higher in the second wave (n=2, 13.33%) as compared to the first wave (n=7, 11.29%). The incidence of premature rupture of membranes (PROM) in the second wave (n=1, 6.66%) was almost similar to the first wave (n=4, 6.45%). There were three (20%) preterm vaginal births in the second wave in comparison to five (8.06%) in the first wave. The number of cesarean sections was higher in the first wave (n=35, 56.45%) in comparison to the second wave (n=5, 33.33%). Normal vaginal births were almost similar in both the first (n=18, 29.03%) and second (n=4, 26.66%).waves. The number of ICU admissions and, hence, disease severity was much higher in the second wave (n=4, 26.66%) as compared to the first wave (n=1, 1.61%) and was significantly associated. Maternal mortality was also significantly associated with the second wave of COVID-19 and there were no maternal deaths during the first wave of COVID-19 disease whereas two (13.33%) maternal deaths occurred in the second wave (Table 4).

**Table 4:** Association of maternal outcomes in first and second waves of COVID-19 during active infection Note: * significant at α=5% PROM: premature rupture of membranes; COVID-19: coronavirus disease 2019

Maternal outcome	First wave of COVID-19		Second wave of COVID-19		P-value
	N=62	%	N=15	%	
Spontaneous abortion	4	6.45	3	20	0.101
Preterm labor	7	11.29	2	13.33	0.82588
PROM	4	6.45	1	6.66	0.97606
Normal vaginal delivery	18	29.03	4	26.66	0.85716
Preterm vaginal delivery	5	8.06	3	20	0.17384
Cesarean section	35	56.45	5	33.33	0.1074
ICU admission	1	1.61	4	26.66	0.00042*
Maternal mortality	0	0	2	13.33	0.00362*

Fever as a complication in the newborn was recorded in seven (11.29%) in the first wave and three (20%) in the second wave. The number of preterm vaginal births was higher in the second wave (n=3, 20%) as compared to the first wave (n=5, 8.06%). One (1.61%) case was admitted to the neonatal intensive care unit (NICU) in the first wave while none were in the second wave. The number of stillbirths was higher in the second wave (n=3, 20%) as compared to the first wave (n=2, 3.22%). All stillbirths in both the first and second waves were of preterm gestation. No newborns tested RT-PCR positive within 24 hours of birth in both waves of the COVID-19 pandemic, whereas day five RT-PCR positivity in the first wave was three (4.83%) and none in the second wave. Exclusive breastfeeding was initiated in all live-born babies within 24 hours of birth in both the first and second waves. No cases of pneumonia or neonatal deaths due to COVID-19 were reported. Among considered fetal and neonatal outcomes, stillbirth and breastfeeding were found to be significantly associated with waves of COVID-19 at α=5% (Table 5).

**Table 5:** Fetal and neonatal outcomes in the first and second waves of COVID-19 pandemic during active infection Note: *significant at α=5% NICU: neonatal intensive care unit; COVID-19: coronavirus disease 2019; RT-PCR: reverse transcription-polymerase chain reaction

Fetal& Neonatal Outcome	First wave of COVID-19	Second wave of COVID-19 (n=15)	P-value
	(n=62)		
Fever	7 (11.29%)	3 (20%)	0.36812
Pneumonia	0 (0%)	0 (0%)	
Preterm birth	5 (8.06%)	3 (20%)	0.17384
NICU admission	1 (1.61%)	0 (0%)	0.61708
Stillbirth	2 (3.22%)	3 (20%)	0.01778*
RT-PCR positive on day 5	3 (4.83%)	0 (0%)	0.3843
Neonatal death	0 (0%)	0 (0%)	
Breastfeeding (In babies delivered at our institute)	56 (90.32%)	9 (60%)	0.00362*

Out of all the patients admitted, 29 (28.71%) delivered at term in the first wave and seven (31.81%) in the second wave. Preterm deliveries were more in the second wave (n=5, 22.72%) as compared to the first wave (n=7, 6.93%). Fifty-nine (58.41%) babies were delivered by Cesarean section in the first wave and seven (31.81%) in the second wave. The incidence of spontaneous abortions was higher in the second wave (n=3, 13.63%) as compared to six (5.94%) in the first. The incidence of stillbirth was also much higher in the second wave as compared to the first wave (n=2, 1.98%). Most of the complications like preterm delivery, spontaneous abortion, and stillbirth took place during the active phase of COVID-19 (Table 6).

**Table 6:** Collective data including long-term follow-up of patients sent for home isolation in COVID-19 pandemic in first and second waves Note: * significant at α=5% COVID-19: coronavirus disease 2019

Pregnancy Outcome	First wave of COVID-19		Second wave of COVID-19		P-value
	N=101	%	N=22	%	
Normal vaginal delivery	29	28.71	07	31.81	0.771
Preterm delivery	07	6.93	05	22.72	0.023*
Cesarean section	59	58.41	07	31.81	0.023*
Spontaneous abortion	06	5.94	03	13.63	0.207
Stillbirth	02	1.98	03	13.63	0.012*

The most common long-term sequelae in both the first and second waves of COVID-19 was fatigue (n=90, 89.1% and n=18, 90%, respectively). Headache was reported in 30 (29.70%) females in the first wave and eight (40%) in the second wave. Loss of taste and smell was found in 15 (14.85%) in the first and five (25%) in the second wave. Cough was reported in 14 (70%) patients in the second wave as compared to 28 (27.72%) in the first wave. Dizziness was seen in two (1.98%) patients in the first and three (15%) in the second wave. Fast breathing was recorded in two (1.98%) and three (15%) patients in the first and second waves of COVID-19, respectively. Joint pain/muscle pain was reported by 55 (54.45%) patients in the first and 10 (50%) patients in the second wave. Diarrhoea was seen in five (4.95%) and two (10%) patients in the first and second waves, respectively. Sleep disturbance was reported by five (4.95%) patients in the first and seven (31.81%) in the second wave. Chest pain was reported by two (1.98%) in the first and four (20%) patients in the second wave. Mood changes were seen in five (4.95%) patients in the first wave of COVID-19 and five (25%) in the second. There were no cases of difficulty in thinking during or after the COVID-19 infection period in the first wave but two (10%) patients with severe infection in the second wave reported this. Long-term sequelae were more marked in the second wave as compared to the first wave. No long-term sequelae were reported in newborns including congenital anomalies in either the first wave or the second wave of COVID-19 (Table 7).

**Table 7:** Long-term sequelae of COVID-19 in first and second waves of COVID-19 pandemic (four or more weeks later)

Outcome	First wave of COVID-19 (n=101)		Second wave of COVID-19 (n=20)	
	No.	%	No.	%
Fatigue	90	89.10	18	90
Headache	30	29.70	8	40
Loss of taste and smell	15	14.85	5	25
Cough	28	27.72	14	70
Fever	22	21.78	5	25
Dizziness	2	1.98	3	15
Fast breathing	2	1.98	3	15
Joint/muscle pain	55	54.45	10	50
Diarrhoea	5	4.95	2	10
Sleep disturbance	5	4.95	7	31.81
Chest pain	2	1.98	4	20
Mood Changes	5	4.95	5	25
Difficulty thinking	0	0.0	2	10
Long-term sequelae in newborn	0	0.0	0	0.0

## Discussion

The number of admissions among pregnant women in the first wave was higher (n=101, 6.99%) as compared to the second wave (n=22, 2.12%). Out of these, the number of admitted asymptomatic patients in the first wave was 73 (72.27%), and in the second wave was 12 (54.54%). Patients with mild disease were 26 (25.74%) in the first and six (27.27%) in the second wave. A study done by Singh et al. reported 91 (65.46%) and 43 (39.09%) asymptomatic and 46 (33.09%) and 53 (48.18%) patients with mild symptoms in the first and second waves, respectively [11].

In our study, in the first wave, only one patient with mild symptoms who underwent Cesarean section for obstetric indication developed severe disease post surgery with SpO2 < 68%; however, the patient recovered by postoperative day six with non-invasive ventilatory support. The reported number of severe to critically ill patients was higher in the second wave (n=4, 18.18%) as compared to the first wave (n=1, 0.99%). The study by Singh et al. reported two (1.44%) and four (3.64%) critically ill patients in the first and second waves, respectively [11]. Mahajan et al. also reported higher rates of severe COVID-19, ICU admission, higher case fatality rate, and maternal mortality in the second wave [12].

There were two maternal deaths in the second wave and none in the first wave in our study. Out of the two maternal deaths, one (4.54%) patient succumbed to death due to severe COVID-19 at 24 weeks of gestational age whereas the other patient was a COVID-19-positive case of antepartum eclampsia at 35 weeks gestational age with pontine hemorrhage with low general condition with no evidence of fever or respiratory symptoms prior to episodes of convulsions and her CT thorax findings were within normal limit. According to the study by Singh et al., there was no maternal mortality in the first wave as compared to four (3.64%) in the second wave [11]. Case fatality as reported by Mahajan et al. was also high in the second wave (n=22, 5.7%) as compared to the first wave (n=8, 0.7%) [12]. 

In our study, the common co-morbidities were anemia, hypertensive disorders, diabetes, and hypothyroidism; however no conclusion could be drawn as to the association of existing co-morbidities with COVID-19, either in the first wave or the second wave as the cases were either asymptomatic or had mild illness with no acute exacerbation during the hospital stay. Besides this, in certain sub-categories, there was no comparison data. All individuals except anemic pregnant females had disease controlled on medication. A higher number of anemic females (n=5, 22.72%) was reported in the second wave as compared to the first wave (n=4, 3.96%), and all were cases of severe anemia.

All anemic females that were admitted were either asymptomatic or had mild symptoms of COVID-19 and none had episodes of acute exacerbation either in the first or the second wave of the COVID-19 pandemic. In the study done by Singh et al., frequently associated comorbidities were hypertensive disorders, diabetes, and anemia; however, no significant difference in the frequency of these comorbidities was noted in the first or the second wave [11]. Mahajan et al. [12] and Mohini et al. [13] also reported similar findings. In our study, the incidence of spontaneous abortions was higher in the second wave (n=3, 20%) as compared to the first wave (n=4, 6.45%). Spontaneous abortions in the second wave were seen in patients with severe hypoxia.

The incidence of preterm vaginal births was higher in the second wave (n=3, 20%) as compared to the first wave (n=5, 8.06%). However, in both the first and second waves, the patients who went into preterm labor had only mild disease; hence, no conclusion could be drawn as to its direct relation with the degree of viremia. No vertical transmission was noted in the study done by Lui et al. [14] and another study done by Zeng et al. [15]. A study by Smith et al. showed a direct attribution to the degree of viremia as the study noted 63.8% of preterm births in the first wave [16]. In the study by Singh et al., the incidence of preterm birth was high in both the first (n=35, 27.78%) and second (n=21, 24.71%) waves [11]. A similar result of a high incidence of preterm birth was also reported in a study done by Chaudhary et al. [17]. However, the cause for high preterm births remains unclear from these studies.

In this study, in the first few admissions in the first wave, PROM occurred in four (6.45%) patients, which gave an impression that SARS-CoV-2 infection was associated with premature rupture of membranes; however, subsequently, no such cases were noted, and no correlation could be drawn from this finding since liquor amnii was not subjected to examination, and newborns of these patients tested negative for COVID-19 disease at the time of birth on RT-PCR examination of nasopharyngeal swab. The incidence of PROM in the second wave (n=1, 6.66%) was almost similar to the first wave with no impact on the newborn. Results of the study done by Mohini et al. were also not statistically significant with 14 (11%) and 10 (16.1%) cases of PROM in the first and second waves, respectively [13].

The number of Cesarean births was higher in the first wave (n=35, 56.45%) in comparison to the second wave (n=5, 33.33%) in our study. All Cesarean sections were performed for obstetric indications and none because of maternal respiratory distress due to COVID-19. In the study by Singh et al., the incidence of Cesarean section was 82 (58.59%) in the first wave and 67 (60.91%) in the second wave, and all Cesarean sections were performed for obstetrical indications only [11].

The number of ICU admissions and hence disease severity was much higher in the second wave (n=4, 26.66%) as compared to the first wave (n=1, 1.61%) in this study. The study by Mohini et al. showed similar results with four (3.1%) and eight (12.9%) ICU admissions in the first and second waves, respectively, which was statistically significant [13].

The number of stillbirths was higher in the second wave (n=3, 20%) as compared to the first wave (n=2, 3.22%). In the first wave, both the patients were only mildly symptomatic for COVID-19 and the cause of stillbirth was predominantly obstetrical, whereas in the second wave, out of the three cases, one was a case of antepartum eclampsia without evidence of severe COVID-19, one was a case of severe anemia with meconium aspiration, and the third stillbirth was seen in a severe SARS-CoV-2 infected patient at 26 weeks gestational age. Thus, severe SARS CoV-2 infection could be associated with stillbirth as seen in our study. In a study by Roohi and Janaki [18], the number of stillbirths was 32 (4%) and 24 (6%) (p = 0.03), and in the study by Singh et al. [11], it was four (2.88%) and two (1.85%) (p=0.699) in the first and second waves, respectively. However, severity of disease and its association with stillbirth has not been mentioned in the above two studies.

Exclusive breastfeeding was initiated in all live-born babies within 24 hours of birth in both the first and second waves. No newborns at birth were reported RT-PCR positive in either the first or second wave groups, whereas day five RT-PCR positivity in first wave group was three (4.83%) and none in the second wave. Similar results were reported in a study done by Wang et al. [19], thus suggestive of no vertical transmission. However, the study by Singh et al. showed two (1.61%) and one (1.18%) positivity in the first and second waves, respectively [11]. Studies done by Dong et al. [20] and Chaudhary et al. [17] also showed possibilities of vertical transmission. Hosieret et al. confirmed SARS-CoV-2 invasion of the placenta, predominantly localized to syncytiotrophoblast cells at the feto-maternal interface of the placenta [21]. Fever in the newborn was recorded in seven (11.29%) in the first wave and three (20%) in the second wave. Only one (1.61%) newborn was admitted to NICU in the first wave and none in the second wave. This is contrary to the study done by Singh et al., where the NICU admission rate was high in both the first and second waves (n=26, 21.31% and n=28, 33.33%, respectively [11]. Also, Allotey et al., in a systemic review, concluded that pregnant females infected with COVID-19 are more likely to give preterm birth and have a higher incidence of neonatal admission to the ICU [9].

No cases of congenitally anomalous babies were reported on long-term follow-ups of patients that were admitted early in pregnancy. No cases of neonatal deaths or pneumonia as a complication were reported in either the first or the second wave of the COVID-19 pandemic in our study. Neonatal death reported in the study done by Singh et al. was three (2.48%) and one (1.18%) in the first and second waves, respectively (p =0.648); however, none of the neonatal deaths were secondary to COVID-19 infection [11]. In the study conducted by Mohini et al., two (1.6%) and four (6.5%) neonatal deaths were reported in the first and second waves, respectively [13]. Although the number of neonatal deaths was more in the second wave, the results were not statistically significant. The most common long-term sequela in both the first and second waves of COVID-19 was fatigue (n=90, 89.1% and n=18, 90%, respectively). Long-term sequelae were more marked in the second wave as compared to the first wave of the COVID-19 pandemic. Most of the symptoms lasted for a period of 60-90 days post infection in both the first and second waves. Patients in the second wave with severe disease had symptoms like fatigue, myalgia, and arthralgia that lasted up to six months. A systematic review and meta-analysis conducted by Han et al. on long-term sequelae of COVID-19 was suggestive of symptoms ranging from six months to one year [22].

Strength of this study

All antenatal patients were followed up for the entire duration of pregnancy and those patients who delivered during the hospital stay were also followed up to study the long-term sequelae in both mother and newborn.

Limitations

Vertical transmission and the relation between preterm labor and SARS-CoV-2 could not be established in our study as amniotic fluid or vaginal secretions were not subjected to RT-PCR tests. Long-term sequelae in babies other than teratogenicity are beyond the scope of this study and need to be studied further.

Clinical implications

Although the overall risks are low, pregnant females, especially those contracting severe illness, are at an even greater risk for ICU admissions and developing fetal complications and even face higher mortality as compared to non-pregnant females. Thus, this group needs special attention in terms of management, vaccination, or future policy making.

Future recommendations

As SARS-CoV-2 infection still continues to affect individuals worldwide, more and more data needs to be pooled in order to form a meta-analysis on this subject.

## Conclusions

In this study, we found that the maximum number of COVID-19-positive pregnant patients in both the first and second waves were either asymptomatic or had mild infection. The second-wave infection was more severe as compared to the first wave in terms of adverse maternal as well as fetal outcomes. No gestational age was an exception to the severity of disease and its adverse feto-maternal outcome. In our study, maternal co-morbidities did not impact the overall outcome. All Cesarean sections were performed for indications other than COVID-19 infection. Long-term sequelae associated with COVID-19 were seen in both groups but more so in the second wave. No long-term sequelae like congenital anomalies in the babies were associated with COVID-19 either in the first or the second wave. 
